# What is an “early palliative care” intervention? A scoping review of controlled studies in oncology

**DOI:** 10.1002/cam4.6490

**Published:** 2023-10-30

**Authors:** Stephan Nadolny, Eva Schildmann, Elena S. Gaßmann, Jan Schildmann

**Affiliations:** ^1^ Institute for History and Ethics of Medicine, Interdisciplinary Center for Health Sciences Martin Luther University Halle‐Wittenberg Halle Germany; ^2^ Institute for Educational and Health‐Care Research in the Health Sector Hochschule Bielefeld—University of Applied Sciences and Arts Bielefeld Germany; ^3^ Department of Palliative Medicine Medical Faculty, University of Augsburg Augsburg Germany; ^4^ Department of Palliative Medicine LMU University Hospital, LMU Munich Munich Germany

**Keywords:** cancer, early integration, early palliative care, oncology, palliative care, scoping review

## Abstract

**Introduction:**

Early palliative care (EPC) has been advocated to improve cancer patients' health. However, EPC differs with regard to its elements and target groups. It is not known which parts of EPC contribute to effectiveness for which patient group. This scoping review provides a structured analysis of EPC interventions and outcome measures.

**Design:**

We searched EMBASE, MEDLINE, CINAHL, and CENTRAL up to February 2022. We included randomized controlled trials (RCT), nonrandomized trials, cohort studies (CS), and controlled before‐after studies of EPC in adult patients in English, Dutch, and German language. Interventions had to be self‐labeled as EPC. Screening and data extraction were performed by two raters. A structured analysis incorporating the TIDieR checklist was performed to describe the elements of the interventions.

**Results:**

We screened 2651 articles, resulting in 40 articles being included: 34 studies were RCT and six studies were CS with a mean sample size of 208 patients. Patients with pancreatic (*n* = 10) and lung cancer (*n* = 9) were most often included. Studies reported different reference points for the onset of EPC such as time after diagnosis of incurable cancer (*n* = 18) or prognosis (*n* = 9). Thirteen studies provided information about elements of EPC and eight studies about the control intervention. Most frequent elements of EPC were symptom management (*n* = 28), case management (*n* = 16), and advance care planning (ACP; *n* = 15). Most frequently reported outcome measures were health‐related quality of life (*n* = 26), symptom intensity (*n* = 6), resource use, and the patient's mood (*n* = 4 each).

**Conclusion:**

The elicited heterogeneity of ECP in combination with deficits of reporting are considerable barriers that should be addressed to further develop effective EPC interventions for different groups of cancer patients.

## 1 INTRODUCTION

Many patients with incurable cancer suffer from a wide range of physical symptoms and encounter psychological and social challenges associated with the disease as well as due to tumor‐specific treatment.^1^, ^2^, ^3^ Palliative care (PC) contributes to the improved health of these patients by addressing their physical, psychosocial, and spiritual needs. While PC traditionally has been offered to patients in the last phase of life this approach has changed over the past few decades. In line with the definition of the World Health Organization,^4^ it is nowadays recommended to offer PC at earlier stages of the disease trajectory and along with anti‐cancer treatment.^5^

The concept of early PC (EPC) reflects this development.^6^, ^7^, ^8^, ^9^ The American Society of Clinical Oncology (ASCO), for example, recommends that patients with advanced cancer diagnosis should be offered EPC within 8 weeks after diagnosis.^5^ Accordingly, EPC targets a broad range of patients, some of whom may only have a life expectancy of several weeks whereas others may have a life expectancy of several years.

Research suggests that EPC can have a positive impact on a range of outcomes, such as patients' quality of life, the severity of the symptoms experienced and even prolongation of life.^10^, ^11^ To date, however, the evidence is limited, since the certainty regarding the elicited positive effects is low to very low.^10^, ^11^, ^12^, ^13^ Moreover, there is lack of consensus regarding the best timing, elements, and mode of EPC.^5^ This is also reflected in a more recent systematic review about EPC in hematological diseases which stresses the need to specify patient groups, the right time to start with early palliative care as well as the elements of care to provide EPC.^12^ In a similar vein, distinguishing different care models to introduce EPC^7^ and identifying those factors of EPC that contribute most to the improvement of the situations of patients with incurable cancer EPC^14^ has been called for.

One reason for the current debate is the fact that EPC is a so‐called complex intervention which means that the intervention encompasses multiple treatment modalities that may contribute to the (different) effect(s).^15^, ^16^, ^17^ Against this background a detailed knowledge about structure, processes, and targets of the intervention is important. However, to the best of our knowledge, no structured analysis of EPC interventions using an established instrument, such as the Template for Intervention Description and Replication (TIDieR), exists.

To be able to develop and evaluate EPC further, it is necessary to collect and critically appraise studies on commonalities and differences regarding the elements of EPC interventions, which possibly contribute to the effects of EPC. Moreover, given the broad range of patients with advanced cancer and their needs, clarification concerning the right type of EPC intervention for a specific patient group is important. Against this background, this scoping review aims to provide a detailed overview of the elements of EPC interventions, target groups, and reported outcome measures that have been used in controlled trials with cancer patients.

## 2 MATERIALS AND METHODS

We conducted a scoping review in a multidisciplinary team (medical ethicists, physicians with an expertise in oncology and PC, and a nursing scientist). We followed the steps described by Arkey & O'Malley^18^: (1) formulating the research question, (2) identifying relevant studies, (3) selecting relevant studies, (4) charting the data, and (5) collating, summarizing, and reporting the results.

We searched the following databases for available evidence up to February 2022:

- MEDLINE via Pubmed
- CINAHL via EBSCO
- CENTRAL via Cochrane Library
- Cochrane Pain, Palliative and Supportive Care Group (PAPAS) Database via Cochrane Library

We also screened the reference lists of all included articles for other relevant studies.

The search terms and combinations for each database were derived from previous reviews about this topic.^7^, ^10^ They are reported in adherence with the PRISMA‐S checklist^19^ (see Data S1). The search terms were subject to internal quality assurance through the application of the Peer Review of Electronic Search Strategies checklist.^20^ Deduplication of the results was performed by Citavi Version 6.6^21^ and Rayyan software.^22^

Studies were included if they were self‐labeled in title or abstract as “early palliative care” or its synonyms, if the respective study population was adult (≥18 years), and if the publication was written in Dutch, English, or German. We chose that language because we are fluent in those languages. A diverse range of study designs was included to gather a broad range of interventions. Randomized controlled trials, nonrandomized trials, cohort studies, and controlled before‐after studies were eligible for inclusion. We included studies conducted in hospitals or outpatient clinics, but we excluded studies focusing only on home care in order to be able to compare the interventions taking place within hospitals and further clinical settings. Control interventions could be either an active intervention designed for the study or usual care. We do not report outcomes in terms of the effectiveness of EPC due to the focus of this review on elements of the intervention and outcome measures.

Two reviewers (EG and SN) independently screened titles and abstracts and subsequently read the full texts of the papers thus identified.^22^ A third author (JS) was consulted in case of dissent or uncertainty. The search flow was visualized with the PRISMA Flowchart.^23^ The data were extracted by EG and SN independently, using a piloted form that included the following items: year of publication, country, setting, study design, funding, population (inclusion and exclusion criteria with a focus on the type and stage of cancer), outcomes and outcome measures. We used the TIDieR for the extraction of the information on the intervention and the control interventions.^24^ We did not perform a risk of bias assessment because the aim of this review was to assess the content of the interventions and not the effectiveness of EPC, which is in line with the process described by Arksey & O'Malley.^18^ We synthesized the data by means of a narrative and tabular overview of the data regarding study design, sample size, country, cancer entity, time until onset of EPC, outcomes measured, and the elements of the interventions. Although this review does not focus on the pooling of outcomes we decided to synthesize these data, because of information on the consistency of the described elements of EPC with the respective goals.

## 3 RESULTS

**FIGURE 1:**
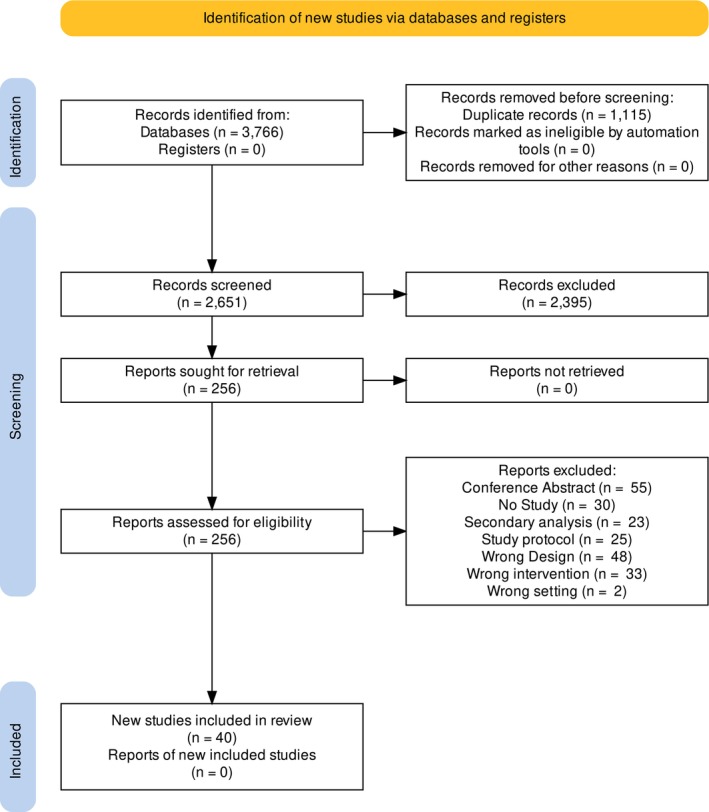
PRISMA flowchart.

The database search yielded 3766 records. After deduplication, 2651 articles remained. Exclusion of 2397 articles was based on the screening of title and abstract. The assessment of the remaining 254 full‐text articles resulted in the inclusion of a total of 40 studies^25^, ^26^, ^27^, ^28^, ^29^, ^30^, ^31^, ^32^, ^33^, ^34^, ^35^, ^36^, ^37^, ^38^, ^39^, ^40^, ^41^, ^42^, ^43^, ^44^, ^45^, ^46^, ^47^, ^48^, ^49^, ^50^, ^51^, ^52^, ^53^, ^54^, ^55^, ^56^, ^57^, ^58^, ^59^, ^60^, ^61^, ^62^, ^63^, ^64^ for synthesis. See Figure 1 for details on the PRISMA Flowchart.

The publication dates ranged from 2009 to 2022. Six studies were cohort studies,^39^, ^41^, ^42^, ^43^, ^47^, ^48^ while the remainder were all randomized controlled trials. Sample sizes ranged from 23^30^ to 969^41^ patients (median = 156, mean = 208). The most prominent study countries were the USA (*n* = 16),^25^, ^26^, ^27^, ^32^, ^33^, ^34^, ^37^, ^39^, ^41^, ^47^, ^50^, ^51^, ^55^, ^56^, ^57^, ^60^ Canada^30^, ^46^, ^63^, ^64^ (*n* = 4) and Italy (*n* = 3).^36^, ^40^, ^49^ Table 1 provides an overview of the included studies.

**TABLE 1:** Overview of included studies.

Study	Study design	Country	Sample size	Setting
Bakitas 2009^25^	RCT	USA	322	Outpatient
Bakitas 2015^26^	RCT	USA	207	Outpatient
Bischoff 2020^27^	CS	USA	60	Outpatient
Brims 2018^28^	RCT	UK, Australia	174	Outpatient
Chen 2022^29^	RCT	China	120	Inpatient
Cusimano 2021^30^	RCT	Canada	23	Outpatient
Do Carmo 2017^31^	RCT	Brazil	63	Both
Dyar 2012^32^	RCT	USA	26	Outpatient
El‐Jawahri 2017^33^	RCT	USA	160	Inpatient
El‐Jawahri 2021^34^	RCT	USA	160	Inpatient
Eychmüller 2021^35^	RCT	Germany	150	Outpatient
Franciosi 2019^36^	RCT	Italy	281	Both
Greer 2022^37^	RCT	USA	120	Outpatient
Groenvold 2017^38^	RCT	Denmark	297	Outpatient
King 2016^39^	CS	USA	207	Outpatient
Maltoni 2016^40^	RCT	Italy	207	Outpatient
May 2015^41^	CS	USA	969	Inpatient
Nakajima 2016^42^	CS	Japan	63	Inpatient
Nieder 2015^43^	CS	Norway	58	Inpatient
Nottelmann 2021^44^	RCT	Denmark	288	Outpatient
Patil 2021^45^	RCT	India	180	Inpatient
Rodin 2020^46^	RCT	Canada	42	Both
Romano 2017^47^	CS	USA	470	Outpatient
Rugno 2014^48^	CS	Brazil	87	Inpatient
Scarpi 2019^49^	RCT	Italy	186	Outpatient
Schenker 2018^50^	RCT	USA	30	Outpatient
Schenker 2021^51^	RCT	USA	672	Outpatient
Slama 2020^52^	RCT	Czech Republic	126	Both
Soto‐Perez‐De‐Ceus 2021^53^	RCT	Mexico	134	Outpatient
Tattersall 2014^54^	RCT	Australia	120	Inpatient
Temel 2010^55^	RCT	USA	151	Outpatient
Temel 2016^56^	RCT	USA	350	Outpatient
Temel 2020^57^	RCT	USA	405	Outpatient
Ullrich 2022^58^	RCT	Germany	80	Outpatient
Vanbutsele 2018^59^	RCT	Belgium	168	Inpatient
Wallen 2012^60^	RCT	USA	152	Inpatient
Woo 2019^61^	RCT	South Korea	288	Outpatient
Zhuang 2018^62^	RCT	China	150	Inpatient
Zimmerman 2014^63^	RCT	Canada	461	Outpatient
Zimmerman 2021^64^	RCT	Canada	110	Outpatient

Abbreviations: CS, cohort study; RCT, randomized controlled trials.

### 3.1 Cancer diagnosis and the onset of early palliative care

Studies included in this review investigated outcomes of EPC for patients with a range of cancer entities, stages of disease, and other characteristics. Most studies included more than one diagnosis, whereas 10 studies^28^, ^29^, ^30^, ^34^, ^37^, ^40^, ^49^, ^50^, ^55^, ^62^ focused on patients with one cancer entity. Patients were most frequently diagnosed with pancreatic cancer (*n* = 10)^31^, ^35^, ^36^, ^40^, ^41^, ^50^, ^52^, ^56^, ^61^, ^64^ and non‐small cell lung cancer (*n* = 9).^25^, ^29^, ^35^, ^36^, ^39^, ^55^, ^56^, ^57^, ^62^ Eight studies^25^, ^29^, ^38^, ^39^, ^45^, ^47^, ^63^, ^64^ used stage III or IV as inclusion criterion for specifying the status of disease, while others described the stage of disease eligible for ECP studies as “advanced” or “incurable.”^26^, ^27^, ^31^, ^32^, ^35^, ^37^, ^40^, ^41^, ^42^, ^44^, ^48^, ^49^, ^50^, ^52^, ^54^, ^55^, ^56^, ^57^, ^58^, ^59^, ^60^, ^61^ Other criteria amenable for EPC in the studies were the line of therapy or treatment^30^, ^33^, ^34^, ^45^, ^52^ or resistance to a certain regime.^31^, ^63^, ^64^ Two studies used a symptom burden over 33% regarding four symptoms, one symptom with at least 50% burden measured by the EORTC‐QLQ‐C30,^38^ or cancer‐related pain higher than 3 measured by the Brief Pain Inventory,^61^ to determine eligibility for the EPC intervention. An Eastern Co‐operative Oncology Group score of 0–2 was often used as an additional criterion for inclusion in the analyzed studies.^28^, ^29^, ^31^, ^35^, ^36^, ^37^, ^40^, ^45^, ^49^, ^50^, ^51^, ^55^, ^56^, ^57^, ^59^, ^62^, ^63^, ^64^ Findings regarding diagnoses and other inclusion criteria are summarized in Table 2.

**TABLE 2:** Overview of cancer diagnoses and additional EPC inclusion criteria.

Study	Entities	Stages or progression^a^	Hematologic/Solid	Further specification	Prognosis (months)	ECOG
Bakitas 2009^25^	Gastrointestinal cancer	Unresectable 3	Solid	Diagnosis withing 8–12 weeks	12	
	NSCLC	4		For breast cancer: estrogen receptor negative, human epidermal growth factor receptor 2 positive		
	Small‐cell lung cancer	3b–4				
	Genitourinary cancer Breast	Extensive				
		4				
		4 and visceral crisis or liver metastasis				
Bakitas 2015^26^	Solid tumor	Advanced	Both		6–24	
	Hematologic malignancies	Advanced stage				
Bischoff 2020^27^	Appendiceal cancer	Metastatic	Solid	Diagnosis after		
	Colorectal cancer					
Brims 2018^28^	Malignant pleural mesothelioma		Solid	Diagnosis within 6 weeks		0–1
Chen 2022^29^	NSCLC	3b–4	Solid	Diagnosis within 8 weeks	>6	0–2
		Metastatic				
Cusimano 2021^30^	Ovarian cancer		Solid	Systematic therapy no more than 30 days prior		
Do Carmo 2017^31^	Breast cancer	Metastatic	Solid	For breast: Me	6–24	0–2
	Ovarian cancer	AND recurrent OR		For ovarian, cervical, endometrial cancer: platinum‐resistant		
	Cervical cancer	Incurable		For neck and head cancer: after radiotherapy failure		
	Endometrial cancer			For prostate cancer: castration‐resistant		
	Neck and head cancer					
	Prostate cancer					
	Bladder cancer					
	Kidney cancer					
	Testicular cancer					
	Penile cancer					
	Lung cancer					
	Colorectal cancer					
	Pancreas cancer					
	Liver cancer					
	Gastric cancer					
	Esophageal cancer					
	Gallbladder cancer					
Dyar 2012^32^	Unspecified cancer	Metastatic	Unclear	Expected hospice referral within 12 months		
El‐Jawahri 2017^33^	Hematologic malignancies	Undergoing HCT	Hematologic	2 weeks after HCT		
El‐Jawahri 2021^34^	Acute myeloid leukemia		Hematologic	Receiving chemotherapy		
Eychmüller 2021^35^	Bladder cancer	Advanced	Solid	Diagnosis within 16 weeks		0–2
	Breast cancer	Metastatic				
	Colorectal cancer					
	NSCLC					
	Pancreatic cancer	Castration‐refractory				
	Prostate cancer					
Franciosi 2019^36^	NSCLC		Solid	Diagnosis within 8 weeks		0–2
	Pancreatic cancer					
	Gastric cancer					
	Biliary tract cancer					
Greer 2022^37^	Breast cancer	Metastatic	Solid	Diagnosis within 8 weeks		0–2
Groenvold 2017^38^	Unspecified cancer	4	Unclear	EORTC QLQ‐C30‐4 symptoms with ≥33% burden		
	CNS cancer	3–4		OR		
				≥1 symptom with ≥50% burden		
King 2016^39^	NSCLC	3b–4	Solid			
	Small‐cell lung cancer	Extensive stage				
Maltoni 2016^40^	Pancreatic cancer	Inoperable	Solid	Diagnosis within 8 weeks	>2	0–2
		AND locally advanced AND/ OR				
		Metastatic				
May 2015^41^	Solid tumor	Metastatic	Both	Admission with 48 h		
	Melanoma					
	Head and neck cancer	Locally advanced				
	Pancreatic cancer					
	Lymphoma	Transplant‐ineligible				
	Multiple myeloma					
	CNS malignancy					
Nakajima 2016^42^	Unclear	Advanced	Unclear			
		OR				
		Recurring				
Nieder 2015^43^	Primary tumors		Solid	Diagnosis and palliative radiotherapy within 12 weeks		
	Lymph node metastases					
	Distant metastases					
Nottelmann 2021^44^	Solid tumors	Metastatic	Solid	Diagnosis within 8 weeks		
		OR				
		Unresectable				
Patil 2021^45^	Head and neck cancer	4	Solid	Planned palliative chemotherapy		0–2
		OR				
		Recurrence				
Rodin 2020^46^	Acute myeloid leukemia		Hematologic	Admission to cancer center within 4 weeks		
	Acute lymphocytic leukemia					
Romano 2017^47^	Solid tumors	4	Solid			
		OR				
		Other advanced				
		AND incurable				
Rugno 2014^48^	Breast cancer	Advanced	Solid			
	Endometrium cancer					
	Ovary cancer					
	Uterine cancer					
	Cervix cancer					
	Vulva cancer					
	Vagina cancer					
Scarpi 2019^49^	Gastric cancer	Inoperable	Solid		>2	0–2
		AND locally advanced				
		AND/OR				
		Metastatic				
Schenker 2018^50^	Pancreatic adenocarcinoma	Locally advanced OR	Solid	Diagnosis within 8 weeks		0–2
		Metastatic				
		OR				
		Borderline resectable				
Schenker 2021^51^	Solid tumors	Metastatic	Solid			0–2
Slama 2020^52^	Lung carcinoma	Advanced	Solid	Noncurative treatment within 12 weeks		
	Pancreatic cancer			For colorectal: third or higher line of systematic therapy		
	Gastric cancer					
	Head and neck cancer					
	Colorectal carcinoma					
Soto‐Perez‐De‐Ceus 2021^53^	Solid tumors		Solid	Diagnosis within 6 weeks		
Tattersall 2014^54^	Solid tumors	Incurable	Solid		<12	
		AND metastatic				
Temel 2010^55^	NSCLC	Metastatic	Solid	Diagnosis within 8 weeks		0–2
Temel 2016^56^	NSCLC	Incurable	Solid	Diagnosis within 8 weeks		0–2
	Small‐cell mesothelioma					
	Pancreatic cancer					
	Esophageal cancer					
	Gastric cancer					
	Hepatobiliary cancer					
Temel 2020^57^	NSCLC	Incurable	Solid	Diagnosis within 8 weeks		0–2
	Small‐cell lung cancer					
	Mesothelioma					
	Non‐colorectal GI cancer					
Ullrich 2022^58^	Unspecified cancer	Incurable	Unclear	Diagnosis within 6–12 weeks		
Vanbutsele 2018^59^	Solid tumors	Advanced	Solid	Diagnosis within 12 weeks	12	0–2
Wallen 2012^60^	Solid tumors	Advanced	Solid	Surgery scheduled		
Woo 2019^61^	Pancreatic cancer	Locally advanced	Solid	Diagnosis within 8 weeks		
	Biliary tract cancer			Cancer‐related pain BPI >3		
				Depression CES‐D > 16		
				Karnofsky performance rating scale ≥50%		
Zhuang 2018^62^	NSCLC		Solid	Diagnosis within 8 weeks		0–2
Zimmerman 2014^63^	Solid tumors	3 with poor prognosis	Solid	For breast and prostate cancer: refractory to hormonal therapy	6–24	0–2
		4				
Zimmerman 2021^64^	Advanced cancer	4		For breast and prostate cancer: refractory to hormonal therapy	≥6	0–2
	Lung and pancreatic cancer	3				

Abbreviations: ECOG, Eastern Co‐operative Oncology Group score; NSLC, non‐small‐cell lung cancer.

^a^ A stage was given if it was stated in the text, otherwise, further descriptions of the progression were reported.

Studies reported different reference points to define the onset of EPC: (a) time after (advanced) cancer diagnosis, (b) estimated prognosis, and (c) other. The majority of those studies used time after diagnosis as a reference point for the onset of EPC with a time span of 8 weeks (*n* = 12),^25^, ^29^, ^36^, ^37^, ^40^, ^44^, ^50^, ^55^, ^56^, ^57^, ^61^, ^62^ 12 weeks (*n* = 4),^25^, ^43^, ^58^, ^59^ 6 weeks (*n* = 2),^28^, ^53^ 90 days (*n* = 1),^27^ and 16 weeks (*n* = 1).^35^ Estimated life expectancy was used as an (additional) reference point for the onset of EPC in nine studies: four studies used a prognosis of 6–24 months,^26^, ^31^, ^63^, ^64^ three studies an estimated life expectancy of 12 months^25^, ^54^, ^59^ and two studies an estimated life expectancy of less than 2 months as inclusion criterion.^40^, ^49^

### 3.2 Outcome measures and elements of the intervention

The reporting of outcome measures in this review is intended to provide information on the correspondence between the described elements of EPC and the respective goals. The most common primary outcomes measured were health‐related quality of life (*n* = 26),^25^, ^26^, ^27^, ^28^, ^32^, ^33^, ^34^, ^36^, ^37^, ^38^, ^40^, ^42^, ^44^, ^45^, ^48^, ^50^, ^51^, ^52^, ^53^, ^54^, ^56^, ^57^, ^59^, ^62^, ^63^, ^64^ symptom intensity^25^, ^26^, ^30^, ^46^, ^54^, ^58^ (*n* = 6), resource use (e.g. treatment costs),^25^, ^26^, ^41^, ^47^ and mood^26^, ^37^, ^48^, ^52^ (*n* = 4 each). Further details about the primary outcomes measured can be found in Table 3.

**TABLE 3:** Primary outcomes measured in the studies.

Study	QoL	Symptom intensity	Resource use	Mood	Trial Outcome Index	Survival	Degree of perceived support	Primary need	Pain	Prognostic awareness	Depression	Treatments	Place of death	Quality of care	Distress
Bakitas 2009^25^	X	X	X												
Bakitas 2015^26^	X	X	X	X		X									
Bischoff 2020^27^	X									X					
Brims 2018^28^	X														
Chen 2022^29^					X										
Cusimano 2021^30^		X									X				
Do Carmo 2017^31^											X				
Dyar 2012^32^	X														
El‐Jawahri 2017^33^	X														
El‐Jawahri 2021^34^	X														
Eychmüller 2021^35^															X
Franciosi 2019^36^	X														
Greer 2022^37^	X			X											
Groenvold 2017^38^	X							X							
King 2016^39^						X									
Maltoni 2016^40^	X														
May 2015^41^			X												
Nakajima 2016^42^	X													X	
Nieder 2015^43^						X									
Nottelmann 2021^44^	X														
Patil 2021^45^	x														
Rodin 2020^46^		X													
Romano 2017^47^			X									X	X		
Rugno 2014^48^	X			X											
Scarpi 2019^49^					X										
Schenker 2018^50^	X														
Schenker 2021^51^	X														
Slama 2020^52^	X			X											
Soto‐Perez‐De‐Ceus 2021^53^	X														
Tattersall 2014^54^	X	X					X								
Temel 2010^55^					X										
Temel 2016^56^	X														
Temel 2020^57^	X														
Ullrich 2022^58^		X													X
Vanbutsele 2018^59^	X														
Wallen 2012^60^									X						
Woo 2019^61^									X		X				
Zhuang 2018^62^	X														
Zimmerman 2014^63^	X														
Zimmerman 2021^64^	X														

Thirteen studies^25^, ^26^, ^31^, ^32^, ^36^, ^40^, ^41^, ^46^, ^50^, ^52^, ^59^, ^61^, ^63^ provided more detailed information about the intervention and underlying theoretical assumptions (see Table 4). In this respect, three studies^26^, ^46^, ^49^ explained the theories regarding the choice of the interventions. Two studies^46^, ^63^ provided a conceptual framework that captures those structural and/process elements that are conceived to contribute to the effects of EPC (so‐called active elements).^15^

The assessment and management of physical (*n* = 28), psychological/emotional/spiritual (*n* = 27), and social (*n* = 21) symptoms or needs are the most common interventions within the context of ECP. Case management or coordination of care (*n* = 16),^25^, ^28^, ^29^, ^35^, ^36^, ^40^, ^43^, ^44^, ^45^, ^47^, ^49^, ^53^, ^55^, ^57^, ^61^, ^62^ goals of care/ACP/assistance with decision‐making (*n* = 15),^29^, ^30^, ^32^, ^34^, ^35^, ^36^, ^39^, ^41^, ^42^, ^44^, ^53^, ^55^, ^57^, ^59^, ^64^ illness understanding or coping (*n* = 15),^33^, ^34^, ^37^, ^39^, ^40^, ^42^, ^44^, ^46^, ^50^, ^51^, ^52^, ^53^, ^55^, ^57^, ^59^ and education (*n* = 6)^25^, ^26^, ^31^, ^46^, ^54^, ^61^ were also often mentioned to be part of EPC in analyzed studies. Three studies provided information on resources (e.g. information leaflet for patients) to support the intervention. Eight studies^33^, ^36^, ^38^, ^40^, ^46^, ^49^, ^61^, ^63^ gave more detailed information on elements of the standard care that constituted the control intervention.^65^ Table 5 summarizes the details of the elements and mode of intervention.

**TABLE 4:** TIDieR checklist elements covered.

Study	Why (goals)?	Why (rationale)?	What (materials)?	What (process)?	Who?	How?	Where?	When?	How much?	Control group intervention sufficiently described?
Bakitas 2009^25^	x		x	x	x	x	x	x	x	
Bakitas 2015^26^	x	x		x	x	x	x	x	x	
Bischoff 2020^27^	x				x	x	x	x	x	
Brims 2018^28^	x				x	x	x	x	x	
Chen 2022^29^	x				x	x	x	x	x	
Cusimano 2021^30^	x				x	x	x	x	x	
Do Carmo 2017^31^	x			x	x	x	x	x	x	
Dyar 2012^32^	x		x	x	x	x			x	
El‐Jawahri 2017^33^	x				x	x	x	x	x	x
El‐Jawahri 2021^34^	x				x	x	x	x	x	
Eychmüller 2021^35^	x	x	x	x	x	x	x	x	x	x
Franciosi 2019^36^	x			x	x	x	x	x	x	x
Greer 2022^37^	x			x	x	x	x	x	x	
Groenvold 2017^38^	x			Deliberately non‐standardized	x	x	x	x	x	x
King 2016^39^	x				x	x	x			
Maltoni 2016^40^	X			x	x	x		x	x	x
May 2015^41^	x			x			x	x		
Nakajima 2016^42^	x				x	x	x	x	x	
Nieder 2015^43^	x				x		x			
Nottelmann 2021^44^	x				x	x	x	x	x	
Patil 2021^45^	x			x	x	x	x	x	x	
Rodin 2020^46^	x	x		x	x	x	x	x	x	x
Romano 2017^47^	x				x			x		
Rugno 2014^48^	x						x			
Scarpi 2019^49^	x	x			x	x	x	x	x	x
Schenker 2018^50^	x			x	x	x	x	x	x	
Schenker 2021^51^	x				x	x	x	x	x	
Slama 2020^52^	x			x	x	x	x	x	x	
Soto‐Perez‐De‐Ceus 2021^53^	x			x	x	x	x	x	x	
Tattersall 2014^54^	x				x	x	x	x	x	
Temel 2010^55^	x				x	x	x	x	x	
Temel 2016^56^	x				x	x	x	x	x	
Temel 2020^57^					x	x	x	x	x	
Ullrich 2022^58^	x				x	x	x	x	x	
Vanbutsele 2018^59^	x			x	x	x	x	x	x	
Wallen 2012^60^	x				x		x	x		
Woo 2019^61^	x			x	x	x	x	x	x	x
Zhuang 2018^62^	x		x		x		x	x	x	
Zimmerman 2014^63^	x			x	x	x	x	x	x	x
Zimmerman 2021^64^	x			x	x	x	x	x	x	

**TABLE 5:** Elements and mode of EPC interventions.

Study	Symptom management (physical)	Symptom management (psychological/ emotional/ spiritual)	Symptom management (social)	Illness understanding/ coping	Goals of care/ ACP	Assistance with decision‐making	Case management	Education	Dosis	Single/ team approach
Bakitas 2009^25^	x	x	x				x	x	1×/month	Team
Bakitas 2015^26^								x	1×/week	Team
Bischoff 2020^27^	x	x							Every 3 months	Unclear
Brims 2018^28^	x	x	x				x		1×/month	Single
Chen 2022^29^	x	x	x						1×/month	Team
Cusimano 2021^30^	x	x	x			x				Single
Do Carmo 2017^31^		x						x	1×/week	Single
Dyar 2012^32^	x	x	x		x				1×/month	Single
El‐Jawahri 2017^33^	x	x		x						Team
El‐Jawahri 2021^34^	x	x		x	x	x			2×/week	Team
Eychmüller 2021^35^	x	x	x		x		x			Team
Franciosi 2019^36^	x	x	x		x	x	x		2×/month	Team
Greer 2022^37^	x	x		x	x	x	x			
Groenvold 2017^38^										Team
King 2016^39^	x	x	x	x	x	x				Single
Maltoni 2016^40^	x	x	x	x			x		1–2×/month	Single
May 2015^41^	x	x	x		x	x				Team
Nakajima 2016^42^	x	x	x	x	x				1×/week	Team
Nieder 2015^43^	x						x		1×/week	Team
Nottelmann 2021^44^	x	x	x	x	x	x	x			Team
Patil 2021^45^	x	x	x				x		1×/month	Team
Rodin 2020^46^	x	x		x				x	1‐2×/week	Team
Romano 2017^47^	x	x	x				x		1×/week	Team
Rugno 2014^48^										Team
Scarpi 2019^49^	x	x	x				x		1‐2×/month	Single
Schenker 2018^50^	x	x	x	x					1×/month	Single
Schenker 2021^51^	x	x	x		x		x		1×/month	Single
Slama 2020^52^	x	x	x	x					Every 6–8 weeks	Single
Soto‐Perez‐De‐Ceus 2021^53^	x			x		x	x		1×/week	Single
Tattersall 2014^54^	x	x	x					x	1×/month	Single
Temel 2010^55^	x	x	x	x	x	x	x			Team
Temel 2016^56^	x	x	x						1×/month	Team
Temel 2020^57^	x	x	x	x		x	x		1/month	Team
Ullrich 2022^58^	x									Single
Vanbutsele 2018^59^	x	x	x	x		x			1×/month	Team
Wallen 2012^60^	x	x								Team
Woo 2019^61^	x	x					x	x		Team
Zhuang 2018^62^	x	x	x				x		1×/month	Team
Zimmerman 2014^63^	x	x	x						1×/week	Team
Zimmerman 2021^64^	x	x	x		x				1×/month	Team

### 3.3 Professional groups and models of EPC interventions

The majority of studies provide information about the professional(s) performing the intervention (*n* = 34). In most cases, a PC physician conducted the intervention alone or in a team (*n* = 27) together with nurses (*n* = 22) and other healthcare providers, such as social workers or psychologists. However, it was unclear whether all nurses or nurse practitioners had a PC specialization. In five studies, a PC or advanced practice nurse was solely responsible for the intervention.^26^, ^32^, ^53^, ^58^, ^59^ The number or frequencies of tasks performed as part of the EPC interventions (e.g. consultations by a PC specialist per month) are reported in 38 studies. 32 studies provide data about the mode (e.g. telephone, face‐to‐face) PC specialists consulted their patients as part of the intervention. Regarding the model of implementation of EPC during the intervention period, four studies describe a *consultative approach* in the hospital, according to which one of the members of the PC team sees the patient and provides advice to the treating oncological team. Six studies describe an approach according to which EPC was *embedded* in an existing interdisciplinary team. The majority of studies (*n* = 30) reported a *solo practice approach* in outpatient clinics, which often includes a strong coordination of care or case management approach (Table 5). Box 1 provides illustrations of the implementation of EPC according to the models of “consultative,” “embedded” or “solo practice.”^66^

BOX 1 **Examples of models of implementation.**

The study by In Slama (2020)^52^ is an example of a *consultative approach* in which a palliative care physician was consulted by the oncology team and visited the patient every 6–8 weeks. He assessed the physical, psychological, and social needs, coping, and need for psychosocial support. The palliative care physicians then gave recommendations for treatment to the oncologists and did not employ care plan changes themselves.

The studies of Vanbutsele (2018)^59^ and Rodin (2020)^46^ are examples of an *embedded approach*. In Vanbutsele (2018),^59^ a palliative care team was introduced early in the disease trajectory and focused strongly on the assessment of physical, psychological and spiritual needs, illness understanding and medical decision‐making. The palliative care team is able to implement care plan changes and is part of the multidisciplinary case conferences. Here, the team works alongside other disciplines with their own competencies regarding decision‐making and treatment. In the study by Rodin (2020),^46^ the intervention consists of psychological and physical components comprising an assessment of physical and psychological needs, education and coping strategies. While the psychological component incorporated 12 educational sessions based on cognitive behavioral therapy performed by the palliative care specialists, the physical component focused on a needs assessment two to three times a week. The usual team takes on the case up to a certain score in the needs burden assessment, while the specialist palliative care team applied symptom management over this value.

The studies of Brims (2018),^28^ Temel (2010),^55^ Zimmerman (2014)^63^ and Zhuang (2018)^62^ are examples of the *solo practice*. In all these studies, patients met with the clinicians in the outpatient setting, which performed physical, psychological and spiritual needs and incorporated care plans or organized further interventions performed by others. The content of the interventions then varies slightly, for example, most studies focus on the assessment and management of the aforementioned needs, while Temel (2010)^55^ also incorporated goals of care planning. Some studies also had an educational component, for example, Bakitas (2009, 2015)^25^, ^26^ and Rodin (2020),^46^ which focused on problem‐solving, symptom management, self‐care, identification and coordination of resources, communication, decision‐making and ACP. The studies also varied regarding the follow‐up mode and frequency. Brims (2018)^28^ and Temel (2010)^55^ have a monthly follow‐up with meetings in the outpatient clinic, while Zimmerman (2014)^63^ did this via telephone without a clear schedule.

## 4 DISCUSSION

This scoping review provides an up‐to‐date comprehensive and structured analysis of existing EPC interventions in oncology. The broad range of studied cancer entities and the stage of cancer reflect the broad target group for EPC. While some elements of EPC are shared by the majority of EPC interventions (e.g. assessment of patients' symptoms), the structured analysis of the interventions shows that there are also many elements of EPC that are only used in a few studies (e.g. the implementation of ACP). The identified lack of information regarding the nature of EPC interventions as well as the lack of information about interventions in the control group make it difficult to identify those elements of EPC which most likely contribute to improvement of health of the heterogeneous group of patients with incurable cancer.

### 4.1 Triggers to initiate EPC in cancer care and the content of the interventions

The trigger to start EPC for most of the patients was a diagnosis of advanced or incurable cancer and/or a limited life expectancy. However, the objective and perceived health situation of patients being offered EPC seems rather heterogeneous.^67^ Given that EPC directed at patients with a life expectancy of less than 6 months likely need a different care approach compared to an intervention directed at patients with a life expectancy of 2 years it seems crucial to specify the triggers as well as the content of specific EPC interventions.^68^, ^69^ The assessment of patients' symptoms and needs is part of most EPC interventions. Interestingly, there is little information about the role of assessment scores and possible cutoff values, which may be used to determine whether and what kind of EPC should be offered to cancer patients at all. Bearing in mind the findings of research using patient‐reported outcomes in oncology, we argue that the rigorous use of PC assessments may pave the way to more individualized EPC interventions.^14^, ^70^, ^71^, ^72^, ^73^

### 4.2 Models of EPC and the reporting of intervention elements

Our review reveals considerable differences in how EPC is provided. EPC often had a strong case management approach, especially in the outpatient clinics. According to such model EPC not only included the assessment of symptoms but also the provision of all forms of relevant care options and coordination of different services. Such a case management approach is different from the “embedded” or “consultative” approaches (see Box 1). The large differences in how EPC is provided underlines the importance of a detailed description of the EPC intervention to be able to implement it on a larger scale, if successful. However, as indicated by use of the TIDieR, (see Table 4) information on the details of EPC interventions is currently available only for a minor part of interventions.

Clarifying core elements of EPC during different stages of incurable cancer seems also important to clarify specific professional requirements including the distinction between when to consult a generalist or specialist in PC.^66^, ^68^ While specialist knowledge may be worthwhile for specific symptomatic treatment, this may be less the case for basic supportive care, such as treatment of pain which is likely to be delivered as part of standard oncology care.^66^

Most studies did not report in detail what has been provided as part of the control intervention. This is of particular relevance for a meaningful interpretation of findings given the fact that PC measures have been introduced at many places as part of standard care for patients with advanced and/or incurable cancer simply^65^ In cases, in which patients in the control group also receive some basic PC, the effects of EPC will probably be underestimated. Knowledge about the measures in the control group is necessary to estimate the effectiveness of the EPC intervention in a real‐world clinical setting.^65^, ^74^

The broad range of target groups and stakeholders in ECP interventions, and the various intervention elements identified in this systematic review clearly show that EPC fulfills the characteristics of “complex interventions”.^15^ Against this background, it comes as some surprise that no logic model or other theoretical account^75^, ^76^ seems to exist for the majority of EPC interventions. We argue that development of conceptual frameworks for particular EPC interventions may contribute to improved outcome research in terms of matching interventions and specific outcomes. In addition, we think the findings of this review and in particular the different elements of EPC, are also relevant from a clinical practice perspective. For example, it allows clinicians to stratify EPC interventions according to the goals that should be achieved for a given patients at a defined point in time in the disease trajectory. This may well differ, as EPC for some patients may be close to supportive care, while for others it may focus on advance care planning. Against this background, a more nuanced approach of EPC interventions may support the choice of which patient should be offered which type of EPC at which point in time.

### 4.3 Limitations

We employed a rigorous search strategy, however, some smaller studies on the topic might have been missed. Although we contacted the study authors in the case of severely missing data, we did not receive responses in all cases. Nonetheless, our results reflect the actual reporting of the studies. We searched only five databases for studies in English, Dutch and German, but there may be additional evidence in other languages as well as databases. In addition, we limited the search to specific quantitative study designs and there is additional evidence in qualitative studies on this topic. From a practice perspective, another limitation is the lack of distinction between primary and specialist palliative care interventions due to the lack of information in the included studies as well as differences in the organization of palliative care in the different countries where the studies were conducted.

## 5 CONCLUSIONS

Overall, a variety of interventions in the field of early palliative care already exist for different entities. They differ in the triggers and the mode of intervention as well as the objectives. There are clear commonalities between specific interventions. However, considerable work has to be done regarding the transparency and comprehensibility of those intervention.

## AUTHOR CONTRIBUTIONS

**Stephan Nadolny:** Conceptualization (supporting); data curation (lead); formal analysis (equal); investigation (equal); methodology (equal); writing – original draft (lead); writing – review and editing (equal). **Eva Schildmann:** Formal analysis (equal); validation (equal); writing – review and editing (equal). **Elena Sophie Gaßmann:** Formal analysis (equal); investigation (equal); writing – review and editing (equal). **Jan Schildmann:** Conceptualization (lead); formal analysis (equal); methodology (equal); project administration (lead); supervision (lead); validation (equal); writing – review and editing (equal).

## FUNDING INFORMATION

This research did not receive any specific grant from funding agencies in the public, commercial, or not‐for‐profit sectors.

## CONFLICT OF INTEREST STATEMENT

The authors declare that they have no known conflict of interest.

## Supporting information


Data S1.

## Data Availability

The data from this review can be obtained upon reasonable request to the corresponding author.
