# Supplementary material for: What is an “early palliative care” intervention? A scoping review of controlled studies in oncology
Source: Cancer Med. 2023 Oct 30;12(23):21335–53. doi: 10.1002/cam4.6490 (PMC10726823; doi:10.1002/cam4.6490)
Supplement: Supplementary file 1 — Data S1. [file CAM4-12-21335-s001.pdf]

Search strategy

Cinahl

#	Query	Results
S1	(((Early OR timely OR proactive) AND ((MH "Palliative Care") OR (MH "Hospice and Palliative Nursing") OR (MH "Terminal Care+") OR (MH "Terminally Ill Patients+"))) OR "early palliative care")	3.435
S2	((MH "randomized controlled trials") OR (MH "double-blind studies") OR (MH "single-blind studies") OR ("MH random assignment") OR (MH "pretest-posttest design+") OR (MH "cluster sample") OR (TI (randomised OR randomized)) OR (AB (random*)) OR (TI (trial)) OR (MH ("sample size") AND AB (assigned OR allocated OR control)) OR (MH ("placebos")) OR (PT (randomized controlled trial)) OR (AB (control W5 group)) OR (MH ("crossover design") OR MH ("comparative studies")) OR (AB (cluster W3 RCT))	984.173
S3	(MH "Nonrandomized Trials") OR (MH "Case Control Studies") OR (MH "Prospective Studies") OR (MH "Concurrent Prospective Studies") OR (MH "Nonconcurrent Prospective Studies") OR (MH "Quasi-Experimental Studies") OR (MH "Interrupted Time Series Analysis") OR (MH "Factorial Design") OR (MH "Controlled Before-After Studies")	580.453
S4	S2 OR S3	1.371.516
S5	S1 AND S4	792

CENTRAL

#	Query	Results
1	MeSH descriptor: [Palliative Care] explode all trees	1.763
2	MeSH descriptor: [Terminal Care] explode all trees	503
3	MeSH descriptor: [Terminally Ill] explode all trees	94
4	(Early OR timely OR proactive)	140.905
5	"Supportive Care"	6.349
6	"Early palliative care"	305
7	#1 OR #2 OR #3 OR #5	8.282
8	#4 AND #7	1.327
9	#6 OR #8	1.530

Pubmed

#	Query	Results
1	(((Early OR Timely OR proactive)) AND (Palliative care[mesh] OR Terminal Care[Mesh] OR Terminally ill[mesh])) OR "early palliative care") AND (cohort studies[mesh:noexp] OR Case-Control Studies[Mesh:noexp] OR Control Groups[Mesh:noexp] OR (case[TIAB] AND control[TIAB]) OR (cases[TIAB] AND controls[TIAB]) OR (cases[TIAB] AND controlled[TIAB]) OR (case[TIAB] AND comparison*[TIAB]) OR (cases[TIAB] AND comparison*[TIAB]) OR control group[TIAB] OR control groups[TIAB] OR Comparative study[PT] OR "before and after"[tiab] OR "before-after"[tiab] OR "CBA"[tiab] OR "CBAs"[tiab] OR Controlled Before-After Studies[mesh] OR Clinical Trial[PT:NoExp] OR clinical trial, phase i[pt] OR clinical trial, phase ii[pt] OR clinical trial, phase iii[pt] OR clinical trial, phase iv[pt] OR controlled clinical trial[pt] OR multicenter study[pt] OR randomized controlled trial[pt] OR Clinical Trials as Topic[mesh:noexp] OR clinical trials, phase i as topic[MeSH Terms:noexp] OR clinical trials, phase ii as topic[MeSH Terms:noexp] OR clinical trials, phase iii as topic[MeSH Terms:noexp] OR clinical trials, phase iv as topic[MeSH Terms:noexp] OR controlled clinical trials as topic[MeSH Terms:noexp] OR randomized controlled trials as topic[MeSH Terms:noexp] OR early termination of clinical trials[MeSH Terms:noexp] OR multicenter studies as topic[MeSH Terms:noexp] OR Double-Blind Method[Mesh] OR ((randomised[TIAB] OR randomized[TIAB]) AND (trial[TIAB] OR trials[tiab])) OR ((single[TIAB] OR double[TIAB] OR doubled[TIAB] OR triple[TIAB] OR tripled[TIAB] OR treble[TIAB] OR treble[TIAB]) AND (blind*[TIAB] OR mask*[TIAB])) OR ("4 arm"[tiab] OR "four arm"[tiab]))	1444
